# Point-by-point compositional analysis for atom probe tomography

**DOI:** 10.1016/j.mex.2014.02.001

**Published:** 2014-03-05

**Authors:** Leigh T. Stephenson, Anna V. Ceguerra, Tong Li, Tanaporn Rojhirunsakool, Soumya Nag, Rajarshi Banerjee, Julie M. Cairney, Simon P. Ringer

**Affiliations:** a Australian Centre for Microscopy & Microanalysis, and School of Aerospace, Mechatronic and Mechanical Engineering, The University of Sydney, NSW 2006, Australia; b Centre for Advanced Research and Technology and Department of Materials Science and Engineering, University of North Texas, Denton, TX, USA

**Keywords:** Atom probe compositional analysis, Atom probe microscopy, Binomial analysis, Nearest neighbours, Compositional analysis

## Abstract

This new alternate approach to data processing for analyses that traditionally employed grid-based counting methods is necessary because it removes a user-imposed coordinate system that not only limits an analysis but also may introduce errors. We have modified the widely used “binomial” analysis for APT data by replacing grid-based counting with coordinate-independent nearest neighbour identification, improving the measurements and the statistics obtained, allowing quantitative analysis of smaller datasets, and datasets from non-dilute solid solutions. It also allows better visualisation of compositional fluctuations in the data. Our modifications include:.

• using spherical *k*-atom blocks identified by each detected atom's first *k* nearest neighbours.
• 3D data visualisation of block composition and nearest neighbour anisotropy.
• using *z*-statistics to directly compare experimental and expected composition curves.

Similar modifications may be made to other grid-based counting analyses (contingency table, Langer-Bar-on-Miller, sinusoidal model) and could be instrumental in developing novel data visualisation options.

## Graphical abstract

**Figure:**
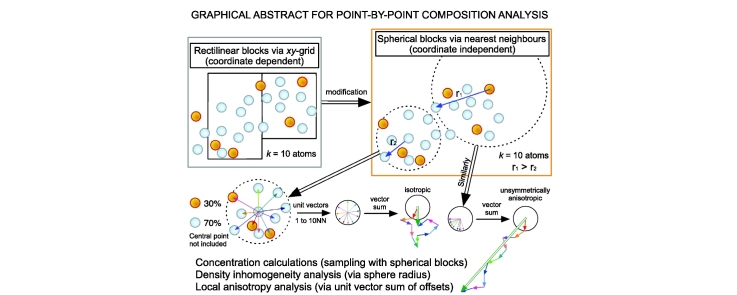

## Method details

### Preparation of material

We demonstrated the new protocol using an atom-probe analysis of a Ni-based super-alloy [1]. In the broader study [2], the Al and Cr segregation is being investigated for its possible association with the nucleation of the γ′-phase precipitates within the matrix γ-phase. The material was processed in the following manner.

• As-cast Ni–8Al–8Cr at.%.
• Solution treatment 1150 °C for 30 min and quenched in liquid nitrogen.
• A further heat treatment at 600 °C for 5 min.
• The atom probe sample was prepared with FEI Nova Nanolab 200 SEM/FIB system.
• 10^6^ atoms were detected using laser-assisted Cameca LEAP 3000XHR.

The operational parameters of the LEAP were a set temperature of 45 K with a pulse rate of 160 kHz and a target evaporation rate of 5 ions per 1000 pulses.

### Construction of spherical blocks

The “binomial analysis” is widely used and provides a relatively rapid test for the presence of non-random compositional fluctuations [3]. The original protocol did this by dividing data into rectilinear *k*-atom blocks, aggregating solute contributions from each block and comparing the one-dimensional compositional histograms with binomial predictions. Later modifications to this protocol achieved many improvements [4], notably by rescaling the imposed (*x*,*y*)-grid to produce *k*-atom blocks that are, on average, more cubic (*z* ≈ *x* = *y*). Highly anisotropic blocks were discounted from the analysis.

Our modifications to the protocol replace the rectilinear *k*-atom blocks with spherical *k*-atom blocks. The spherical blocks increase in radius as *k* is increased. This approach is similar to an earlier protocol to the calculations of atomic concentrations made on the atomic scale (concerning nearest neighbour shells) [5].

The user must select the parameter *k* while being mindful of the minimum size of nanostructural features that can be reliably discerned with this *k*-atom block. Features smaller that these blocks would be smeared with surrounding matrix. Reducing the value of *k* can increase spatial sensitivity but if *k* is too small the local concentrations are computed with smaller samples leading to larger measurement errors.

For each atom, an in-house produced algorithm (employed for earlier studies [6]) was used to perform the following steps.

1. The 1st to the *k*th nearest neighbours were identified and the corresponding spherical coordinates offsets (*r*, *θ*, *ϕ*) were stored (as 32-bit floating point numbers).
2. The chemical identity of the 1st to the *k*th nearest neighbour was also stored (as an 8-bit integer).
3. Using this stored data, the atomic concentration around each atom was calculated.
4. The central point of each block (the origin atom) was excluded from the compositional calculations.

These steps are illustrated in Graphical Abstract in which two spherical blocks are shown, including one in which the atoms have an anisotropic distribution. Dealing with this issue is discussed in section 2B.

Operational speed would be improved by random sampling the data points to serve as central points, or otherwise artificially seeding random central points within the data. Random sampling was not employed as the implementation of the current algorithm was thought to provide a local, complete analysis with data visualisations that have a one-to-one relationship with the original detected atoms. However, random sampling (by either method) would also save storage space. The above protocol stores 13*k* bytes for each atom. In this case we identified up to 1000NN for *all* atoms; the associated information is 812.5 times the size of the original data file.

Each *k*-atom block contributed its atomic concentration to the one-dimensional compositional histogram. In a complete analysis (no random sampling), there are approximately *k* times as many compositional calculations (blocks) in the new analysis as there would be for a standard grid-based method. Many of these blocks spatially overlap but this will not affect the analysis. For instance, if the blocks were randomly sampled so that the blocks used for the concentration calculations do not have significant overlap, the concentrations would still be drawn from the same distribution as the entire ensemble of measurements. In other words, the analysis is not sample dependent and so the analysis is not dependent upon spatial overlap of measurements.

### Removal of low-density and anisotropic blocks

Some blocks, and their contribution to the analysis, were discounted in two cases:

1. spherical blocks that were too large, which was indicative of low-density regions; or
2. blocks where the *k*-atom nearest neighbour distribution was too anisotropic.

**Fig. 1:**
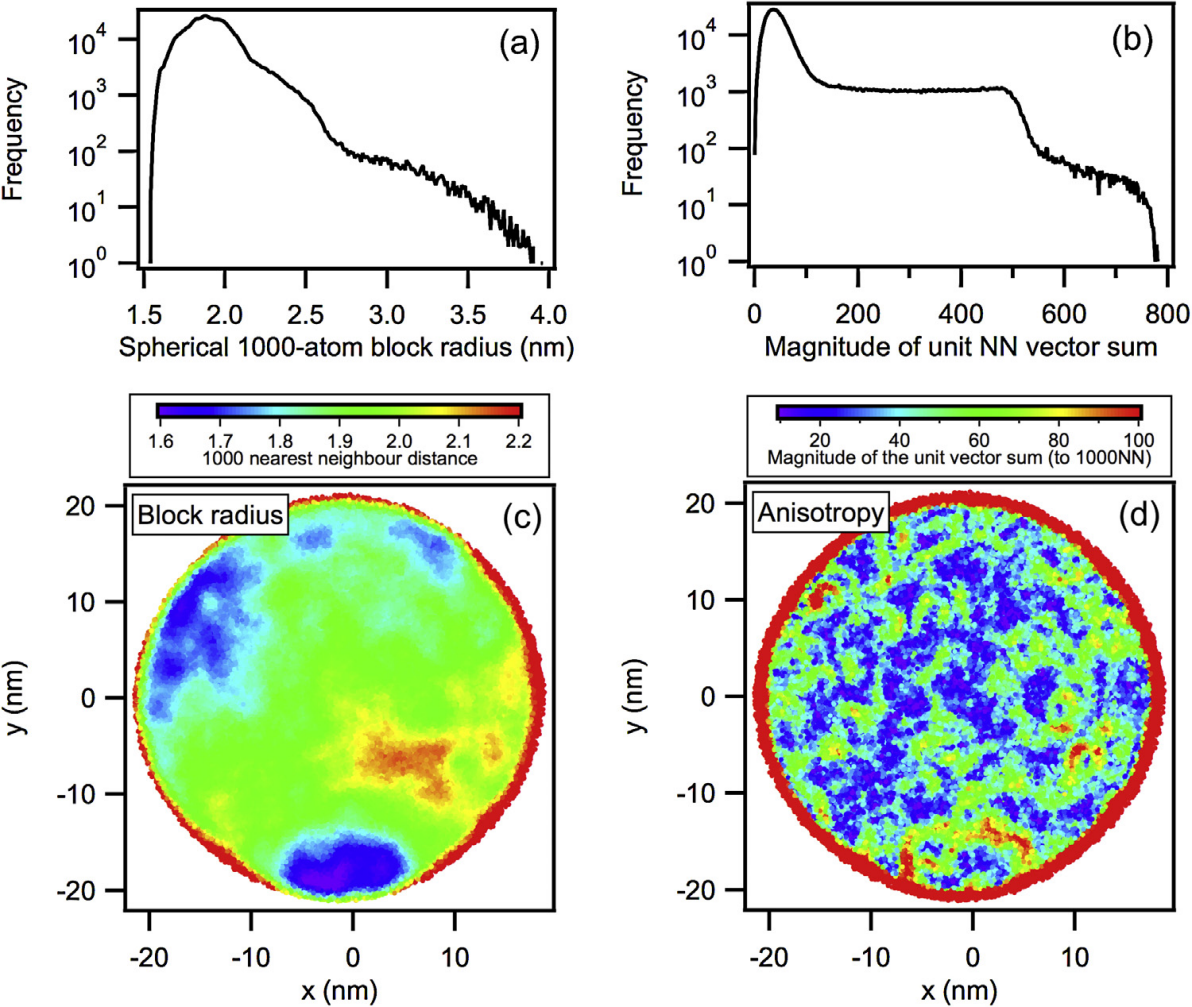
Both the 1000NN radial block size (a) and 1000NN unit vector (b) were calculated and appropriate maximum thresholds were chosen at curve turning points (if *r*_block_ > 2.2 nm or vector sum > 100 this data was discarded). Note that the anisotropy visualisation (d) tracks changes within the data density visualised via the 1000NN block radius (c). In both maps, red marks excessively large radius values or vector sums.

First, we removed regions of abnormally low density: crystallographic poles, detector blind spot and reconstruction surface (see e.g. [6]). This is equivalent to removing grid-based blocks that are too stretched in the *z*-dimension. Fig. 1(a) and (c) visualises the 1000NN distance distribution and a corresponding map. Low-density regions (high 1000NN distances) were evident along the edge of the data and internally in one zone. For the example in this paper, the blocks calculated are between 1.5 nm and 4.0 nm although no block larger than 2.2 nm was used in the quantitative assessment. The distribution tail extends into having many large distances that in Fig. 1(c) are visualised in red.

Second, we calculated the sum of the *k* nearest neighbour unit vectors for all data points so that we could identify atoms nearest to voids (reconstruction surfaces and low density artefacts) and also any atoms that are near to boundaries between phases of different compositions that, if used in a compositional analysis, may blur the composition between mixed density distributions. Nearest neighbour vectors between all atoms in a block were summed to assess the block's anisotropy. Note that for this calculation the *r* spherical coordinate was discarded as only the unit vector was used. The Graphical Abstract illustrates this sum for two data points that have the same atomic concentration. When compiled into a histogram, the distribution of the magnitude of the unit vector sum has a tail that consists almost entirely of data points that are near density fluctuations (data/no-data or high-density/low-density data interfaces).

Fig. 1(b) and (d) demonstrates this data quality assessment for the super-alloy data. The anisotropy can be more effectively used to highlight edges of the data set than the nearest neighbour distance (as used previously, e.g. [6,7]). It also identifies boundaries between regions of different densities but only on a large scale in this case (1000NN distances ≈ 1.5–2.2+ nm).

### Statistical treatments

We calculated two-sample *z*-statistics for each histogram bin comparing the experimental distribution with an expected random distribution. We approximated the standard deviation of the frequency *f*_*b*_ at a particular bin *b* to be

$$\sigma_{b}=\sqrt{f_{b}\left(1-\frac{f_{b}}{N_{\text{blocks}}}\right)}$$

using a Bernoulli trial model described in [8] where the histogram bins are categories for the counting trials of each block and *N*_blocks_ is the integrated sum of the histogram, i.e. the number of blocks. Solute (Al or Cr) composition data was calculated for the experimental data and randomly labelled data (where the list of chemical labels corresponding to the point data is randomly shuffled). Concentrations from randomly labelled data were used as the random comparator instead of a theoretical binomial distribution. Multiple *z*-statistics comparing bin measurements between the two measured histograms were calculated by

$$z_{b}=\frac{f_{b,1}-f_{b,2}}{\sigma_{b,1}^{2}+\sigma_{b,2}^{2}}\text{.}$$

In calculating both the experimental and randomly labelled curves, we discarded data contributing to the Al% and Cr% distributions that corresponded to a 1000NN unit vector sum of more than 100 and/or a block size with a radius of more than 2.2 nm.

**Fig. 2:**
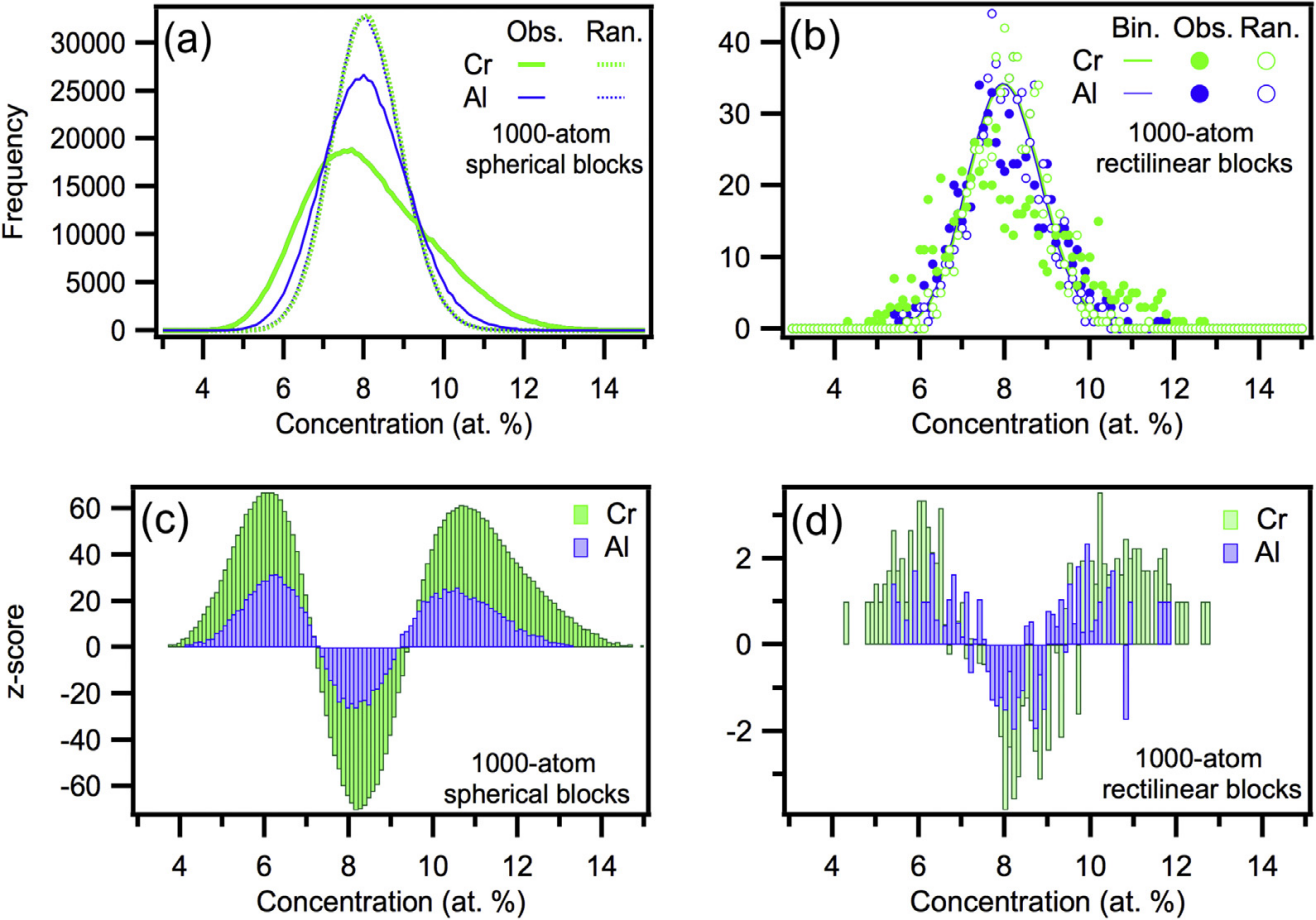
Significant and obvious deviations from the randomly labelled frequency curves were observed for both solute species. The difference was much more distinct in the analysis using many more spherical blocks (a and c) compared to the analysis using the comparatively few rectilinear blocks (b and d). The randomly labelled frequency curves for the analysis using spherical blocks very closely matches a binomial distribution as expected (not shown in (a) for the overlap).

Fig. 2(a) demonstrates the statistical improvements of the new method (using many spherical blocks) over the old protocol (using few rectilinear blocks) to assess the existence of non-random solute segregation within the region of the analysed super-alloy. The distributions have less noise than for the conventional binomial analysis (Fig. 2(b)) and the randomly labelled data for Al and Cr better approach a theoretical binomial distribution. Also, due to the large increase in *N*_blocks_ compared to conventional analysis, Fig. 2(c) shows that the *z*-score curve is less noisy and offers incontrovertible evidence of significant non-random segregation. The Variation protocol [9] calculates the area under the difference curve between normalised distributions to assess the variation amount, producing similar curves to the *z*-score plots. This variation or the coefficient of contingency [4] could be used to characterise the absolute deviation from expected (a binomial distribution or concentration curves calculated from randomly labelled data).

**Fig. 3:**
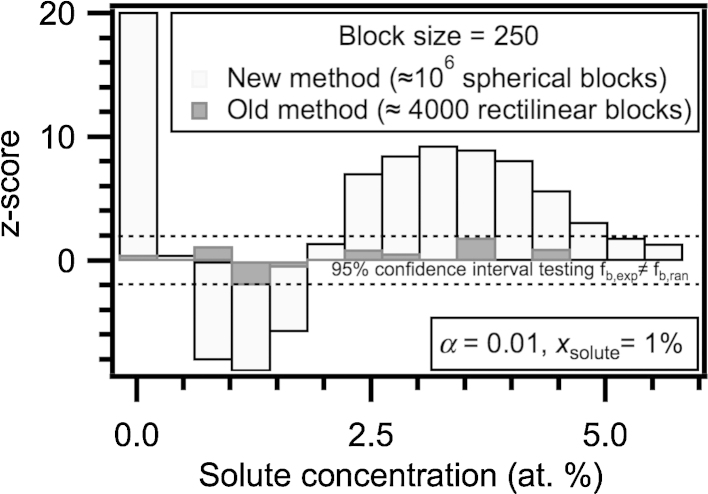
The old and new protocols were trialled upon an APM data simulation corresponding to a low-solute alloy with a very small amount of short-range order (but nonetheless non-random). Only by the new protocol was the data evaluated as significantly non-random. We attribute this to be mainly due to the many more atomic concentration measurements that have been calculated.

Fig. 3 demonstrates how a test for the presence of non-random concentrations benefits from this increase in block number; slightly non-random data (alpha = 0.01) was simulated using techniques described in [10]. The simulated data was found to be non-random by the new protocol within a 95% confidence interval. The old protocol using few rectilinear blocks did not display this sensitivity and assessed the data as random. This difference using spherical blocks is likely to be mostly due to the increased sample size (more concentration calculations) but could also be because the calculations were coordinate independent and more able to resolve the nanostructural fluctuations.

### Chemical visualisation

**Fig. 4:**
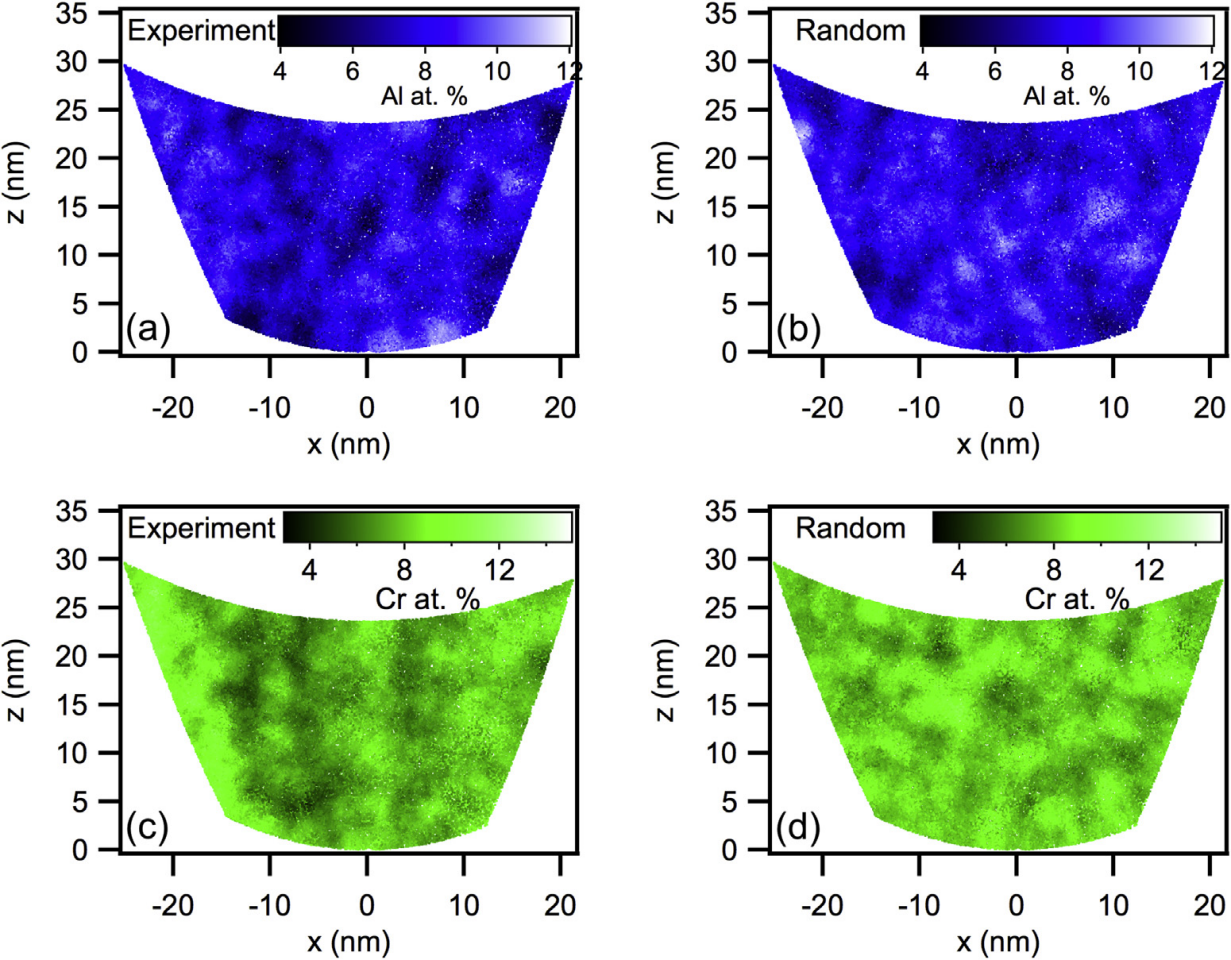
A non-random decomposition was visually observed in both the Al (a vs. b) and Cr (c vs. d) segregation. Solute segregation on a finer scale may be better assessed using a smaller block size (much smaller than *k* = 1000 as in this case). Conversely, solute segregation found using *k* = 1000 on the scale *r*_block_ ≈ 2 nm may be otherwise indiscernible using scales of smaller block sizes.

Fig. 4 demonstrates how the concentrations calculated using the spherical blocks could be directly visualised in three dimensions on a point-by-point basis. This is useful for presenting the data in a meaningful way that has direct connection to quantitative analysis.

## Additional information

### Background information

Atom probe microscopy (APM) is an advanced microanalysis technique that combines position-sensitive ion detection with time-of-flight mass spectroscopy. Amongst other things, this microscope provides atomic-resolution tomographic reconstructions of materials. Individual atoms of the specimen are ionised and accelerated towards a position-sensitive detector. Each detected ion is represented by a chemically labelled data-point, which can be reconstructed to form real-space 3D images and this is referred to as atom probe tomography (APT). APT investigations require sophisticated and computationally intensive data mining and analysis techniques to establish new material science knowledge from the datasets, which may contain 10s of millions of atoms. Amongst the simplest of analyses are those that calculate local solute concentrations, or “composition”.

## References

[1] Reed R.C. (2006). The Super-alloys.

[2] Rojhirunsakool T., Meher S., Hwang J.Y., Nag S., Tiley J., Banerjee R. (2013). Influence of composition on monomodal versus multimodal γ′ precipitation in Ni–Al–Cr alloys. J. Mater. Sci..

[3] Hetherington M.G., Miller M.K. (1989). Some aspects of the measurement of composition in the atom probe. J. Phys..

[4] Moody M.P., Stephenson L.T., Ceguerra A.V., Ringer S.P. (2008). Quantitative binomial distribution analyses of nanoscale like-solute atom clustering and segregation in atom probe tomography data. Microsc. Res. Tech. (MRT).

[5] Hyde J.M., Cerezo A., Williams T.J. (2009). Statistical analysis of atom probe data: detecting the early stages of solute clustering and/or co-segregation. Ultramicroscopy.

[6] Stephenson L.T., Moody Mi.P., Liddicoat P.V., Ringer S.P. (2007). New techniques for the analysis of fine-scaled clustering phenomena within atom probe tomography APT data. Microsc. Microanal..

[7] Shariq A., Al-Kassab T., Kirchheim R., Schwarz R.B. (2010). Exploring the next neighbourhood relationship in amorphous alloys utilizing atom probe tomography. Ultramicroscopy.

[8] Ceguerra A.V., Moody M.P., Stephenson L.T., Marceau R.K.W., Ringer S.P. (2010). A three-dimensional Markov field approach for the analysis of atomic clustering in atom probe data. Philos. Mag..

[9] Auger P., Menand A., Blavette D. (1988). Statistical analysis of atom-probe data (II): theoretical frequency distributions for periodic fluctuations and some applications. J. Phys..

[10] Ceguerra A.V., Moody M.P., Powles R.C., Petersen T.C., Marceau R.K.W., Ringer S.P. (2012). Short-range order in multi-component materials. Acta Crystallogr. A.

